# Negative BOLD in default-mode structures measured with EEG-MREG is larger in temporal than extra-temporal epileptic spikes

**DOI:** 10.3389/fnins.2014.00335

**Published:** 2014-11-18

**Authors:** Julia Jacobs, Antonia Menzel, Georgia Ramantani, Katharina Körbl, Jakob Assländer, Andreas Schulze-Bonhage, Jürgen Hennig, Pierre LeVan

**Affiliations:** ^1^Department of Neuropediatrics and Muscular Diseases, University Medical Center FreiburgFreiburg, Germany; ^2^Epilepsy Center, University Medical Center FreiburgFreiburg, Germany; ^3^Medical PhysicsFreiburg, Germany

**Keywords:** fast fMRI, default mode, epileptic spikes, refractory epilepsy, EEG-fMRI

## Abstract

**Introduction:** EEG-fMRI detects BOLD changes associated with epileptic interictal discharges (IED) and can identify epileptogenic networks in epilepsy patients. Besides positive BOLD changes, negative BOLD changes have sometimes been observed in the default-mode network, particularly using group analysis. A new fast fMRI sequence called MREG (Magnetic Resonance Encephalography) shows increased sensitivity to detect IED-related BOLD changes compared to the conventional EPI sequence, including frequent occurrence of negative BOLD responses in the DMN. The present study quantifies the concordance between the DMN and negative BOLD related to IEDs of temporal and extra-temporal origin.

**Methods:** Focal epilepsy patients underwent simultaneous EEG-MREG. Areas of overlap were calculated between DMN regions, defined as precuneus, posterior cingulate, bilateral inferior parietal and mesial prefrontal cortices according to a standardized atlas, and significant negative BOLD changes revealed by an event-related analysis based on the timings of IED seen on EEG. Correlation between IED number/lobe of origin and the overlap were calculated.

**Results:** 15 patients were analyzed, some showing IED over more than one location resulting in 30 different IED types. The average overlap between negative BOLD and DMN was significantly larger in temporal (23.7 ± 19.6 cm^3^) than extra-temporal IEDs (7.4 ± 5.1 cm^3^, *p* = 0.008). There was no significant correlation between the number of IEDs and the overlap between DMN structures and negative BOLD areas.

**Discussion:** MREG results in an increased sensitivity to detect negative BOLD responses related to focal IED in single patients, with responses often occurring in DMN regions. In patients with high overlap with the DMN, this suggests that epileptic IEDs may be associated with a brief decrease in attention and cognitive ability. Interestingly this observation was not dependent on the frequency of IED but more common in IED of temporal origin.

## Introduction

EEG-fMRI is a non-invasive method to identify epileptic networks activated by IEDs in patients with focal and generalized epilepsy (Gotman et al., 2006; Grouiller et al., 2011). In focal epilepsy, IED-related positive BOLD changes are found in the area of IED origin in the majority of patients (Moeller et al., 2008; Tyvaert et al., 2008). Moreover, positive BOLD changes could be found within or in the vicinity of epileptogenic lesions such as focal cortical dysplasia, nodular heterotopia and mesial temporal sclerosis (Kobayashi et al., 2006b; Jacobs et al., 2007). Zijlmans and colleagues showed that EEG-fMRI can be a useful additional diagnostic tool in the presurgical evaluation of patients with refractory epilepsy by improving the identification of patients suitable for surgery (Zijlmans et al., 2007). This observation was confirmed by a study providing evidence that the surgical removal of the area with the strongest positive BOLD is correlated with a good postsurgical seizure outcome (Thornton et al., 2010; An et al., 2013). Thus, there is strong evidence that BOLD changes related to epileptic spikes are able to identify epileptic networks and EEG-fMRI has a large potential as a diagnostic tool in epilepsy.

Additionally to positive BOLD changes, negative BOLD changes also called deactivations are observed related to IEDs (Archer et al., 2003; Jacobs et al., 2007; Moeller et al., 2008; An et al., 2013). Mechanisms of negative BOLD changes are less well understood in general and their interpretation is subject to debate in patients with epilepsy. Some negative BOLD changes have been observed as focal changes with a close relationship with the spike origin (Jacobs et al., 2007, 2009), but in the majority of cases they are rather widespread and distant (Kobayashi et al., 2006b; Laufs et al., 2007).

An improved understanding of negative BOLD responses is important to facilitate the interpretation of BOLD responses in a clinical setting. Moreover negative BOLD responses may provide additional information about the effect of IEDs on the patient's brain. Many of the observed negative BOLD responses occur in the precuneus, posterior cingulate, bilateral inferior parietal and mesial prefrontal cortices, structures which are known to be part of the default mode network (DMN) (Archer et al., 2003; Gotman et al., 2005). This network was first described in PET studies. Its structures are most active during periods of relaxed wakefulness and their activity is reduced during specific tasks (Mazoyer et al., 2001; Raichle et al., 2001). Strong changes within the DMN have been shown in patients during coma and anesthesia supporting its importance for consciousness (Laureys et al., 2004).

In epilepsy, the strongest negative BOLD in the DMN was observed during absence seizures in idiopathic generalized epilepsy (Moeller et al., 2008; Berman et al., 2010) or following generalized epileptic discharges (Gotman et al., 2005). In patients with focal epilepsy, group analyses reliably detect changes in the DMN (Laufs et al., 2007) and several studies report on negative BOLD in the DMN in some of their individual patients (Fahoum et al., 2012, 2013). In contrast to absence seizures, a change of consciousness is usually not observed in association with focal interictal spikes. Some studies however suggest that focal IEDs in around 50% of patients are associated with a transitory cogitive impairment (TCI) (Binnie, 2003). In focal IED this impairment is especially visible during complex tasks and has most often been shown during studies assessing language or working memory (Hutt and Gilbert, 1980; Aarts et al., 1984). In line with the idea that IEDs can affect cognition the negative BOLD in DMN related to interictal spikes has been interpreted as an indication that focal IEDs may interfere with networks of normal attention (Gotman et al., 2005; Laufs et al., 2007). Interestingly, the observed changes were more prominent during spikes of temporal than extra-temporal origin (Jacobs et al., 2009), which is in line with the more prominent alteration of consiousness during temporal than neocortical seizures. The interference level caused by focal IEDs is probably below the threshold necessary for clinical observation of altered consiousness, but it may still have a long-term influence on the cognitive performance of affected patients.

Recently a number of fast fMRI sequences have been developed (Lin et al., 2006; Feinberg et al., 2010; Posse, 2012). Magnetic Resonance Encephalography is one which allows whole-brain imaging with a temporal resolution of 100 ms (Zahneisen et al., 2012; Assländer et al., 2013). The increased temporal resolution not only improves the tracking of fast artifacts and brain activity, but it also increases sensitivity of functional imaging by recording an increased number of images during each hemodynamic response. Our group could demonstrate that this results in a higher sensitivity to detect IED-associated BOLD changes in focal epilepsy (Jacobs et al., 2014). Moreover, the increased sensitivity resulted in a frequent detection of negative BOLD changes in the DMN even without performing group analysis. It is thus possible to assess the alterations in the DMN associated with IEDs for every single patient, which may be the key to a better understanding of the clinical importance of this phenomenon. The present study aims to quantify negative BOLD in the DMN with the hypothesis that the amount of interference may be dependent on the region of IED generation or the frequency of IEDs observed in each patient.

## Materials and methods

### Patients

Patients with focal epilepsies who were admitted to the Epilepsy Centre Freiburg were included in this study. All patients signed informed consent and the study was approved by the Research Ethics Committee of the University of Freiburg.

EEG-fMRI data were only acquired in patients who fulfilled the following criteria:

1. ability to stay calmly in the MRI scanner over a period of 1 h and
2. frequent IEDs (>10 in 60 min) recorded on routine EEG outside the scanner.

All patients underwent scanning with the EEG-MREG sequence for 20–40 min depending on ability to cooperate.

### Data acquisition

A 64-channel scalp EEG was continuously recorded inside the MRI scanner (3-Tesla Trio Tim, Siemens Healthcare, Erlangen, Germany) with a reference located between Fz and Cz. Sintered Ag/AgCl ring electrodes were attached using a “BrainCap” (Easycap, Herrsching, Germany), which is part of the MR-compatible EEG recording system “BrainAmp-MR” (Brain Products, Munich, Germany). Electrode impedances were kept below 15 kΩ. An electrode was placed perivertebrally on the left for acquisition of the electrocardiogram. Data was transmitted from the amplifier (5 kHz sampling rate synchronized with the 10 MHz scanner clock, 0.016–250 Hz band-pass filter) via an optic fiber cable to a computer located outside the scanner room (Mandelkow et al., 2006). During the whole measurement, the patient's respiration and ECG were monitored with the physiological monitoring unit (pneumatic breathing belt, ECG electrodes) of the MRI scanner (3-Tesla Trio Tim, Siemens Healthcare, Erlangen, Germany).

A 3D, T1-weighted anatomical acquisition (MPRAGE, *TR* = 2200 ms, *TE* = 2.15 ms, FOV = 256 mm, 256 × 256 matrix, 160 sagittal slices, 1 mm slice thickness) was performed for co-registration with the functional images. This was followed by the fMRI acquisition using 3D MREG. Acquisition was performed with the following parameters (*TR* = 100 ms, *TE* = 20 ms, FOV = 192–256 mm, 64 × 64 × 64 matrix, flip angle = 15°, 12,800 volumes, total acquisition time 21:20 min (Zahneisen et al., 2012).

### EEG processing

Gradient artifacts were corrected offline by an averaged artifact subtraction method (Allen et al., 2000). The pulse artifact was then also corrected using averaged artifact subtraction (Allen et al., 1998), followed by an Independent Component Analysis-based procedure to remove residual noise (Srivastava et al., 2005; Debener et al., 2007).

IEDs were marked by two independent reviewers (Julia Jacobs and Katharina Körbl) and were classified into distinct types for each patient according to spatial distribution and morphology (if more than one type was present), verifying that they were similar to epileptic discharges recorded in routine EEG outside the scanner. IED-like transients occurring in a window of 150–500 ms following the QRS complex in the ECG were not marked to avoid including residual ballistocardiographic (BCG) artifact in the analysis (Flanagan et al., 2009; Ertl et al., 2010). EEG quality was considered as appropriate if it allowed the identification of IED types seen in the routine clinical EEG. All IEDs were classified according to their focal distribution at the time of the scan, according to whether they derived from temporal or extra-temporal origin.

### MREG analysis

fMRI images were reconstructed from the raw MREG data (Hugger et al., 2011) and then motion corrected and smoothed (Gaussian kernel, FWHM = 6 mm) using FSL software (http://fsl.fmrib.ox.ac.uk/fsl/fslwiki/) (Smith et al., 2004). Data was then analyzed as an event-related design in the general linear model (GLM) framework using fMRIstat software (Worsley et al., 2002). The noise term in the GLM was modeled as a 5th-order autoregressive (AR) process to account for additional autocorrelations originating from the higher temporal resolution (Worsley et al., 2002; Posse, 2012). The order of the AR model had been determined in a recent study on patients with epilepsy (Jacobs et al., 2014).

IEDs with distinct spatial distribution were analyzed as separate regressors. Motion parameters obtained from the motion correction step and cardio-respiratory regressors based the synchronized recording of the physiological unit of the MRI scanner (Glover et al., 2000) were used as confounds in the model. Four separate event-related analyses were conducted, using HRFs consisting of a single gamma function peaking at 3, 5, 7, or 9 s after the event. This allowed some variation in the latency of the BOLD response while retaining information about its expected shape (Bagshaw et al., 2004). A BOLD response was considered statistically significant if it was significant in any of the 4 analyses. For visualization purposes, a single combined map was thus generated from the highest absolute value for each voxel among the four t-maps. Significant responses were defined as 7 or more contiguous voxels with |t| > 3.5 (*p* = 0.05), corrected for multiple comparisons (Worsley et al., 2002) at the cluster level (Friston et al., 1993) with an additional Bonferroni correction due to the 4 analyses.

### Overlap between the default-mode network and negative BOLD

The following brain regions were considered to be part of the default-mode network: precuneus, posterior cingulate, bilateral inferior parietal and mesial prefrontal cortices (Raichle et al., 2001; Gotman et al., 2005; Laufs et al., 2007). A spatial template of the default-mode network was thus created from those regions as defined in the automatic anatomical labeling (AAL) atlas (Tzourio-Mazoyer et al., 2002). According to the names given in the AAL atlas the following regions were included in the analysis:

- medial prefrontal regions: frontal superior orbital, frontal superior medial, frontal medial orbital, rectus, cingulum anterior
- lateral inferior parietal regions: parietal inferior, angular, supramarginal
- posterior cingulate: *cingulum posterior*
- precuneus: *precuneus*

The atlas was co-registered to each patient's anatomical image, resulting in individual default-mode network templates on which clusters of significant IED-related negative BOLD responses were overlaid.

For each patient, the volume of the default-mode templates, regions of significant negative BOLD responses, and overlap between the two were used to generate the following two measures:

- Percentage of the overall negative BOLD associated with a given IED type, which is located within the structures of the default-mode network
- Percentage of default-mode structures covered by significant negative BOLD changes

The calculated percentages of overlap were then correlated with the number of IED for each IED type using a Spearman correlation. Percentage of overlap was compared for temporal vs. extra-temporal IED using a *t*-test. Significance level for both tests was *p* < 0.05.

## Results

### Patients

Fifteen consecutive patients were included. Six patients showed one, four patients two, four patients three and one patient four distinct IED types. Thus, a total of 30 distinct IED types could be analyzed in this study, 12 of which were classified as temporal IED. Clinical details of all patients are given in Table 1.

**Table 1:** **Clinical information**.

**Patient**	**Age**	**m/f**	**Age of Onset**	**Epilepsy classification**	**Seizure types**	**MRI**	**AED**
1	26	m	7y	Structural	CPS	Hypothalamic harmatoma	LEV, OXC, LCM
2	36	m	7y	Structural TLE	CPS	MTS R	LTG, LCM
3	17	m	13y	Unclear	SPS/CPS GTCS	Normal	LTG, OXC
4	17	f	16y	Structural TLE	CPS	Unclear mass in the L superior T gyrus	OXC
5	27	m	11y	Structural FLE	SPS/CPS GTCS	Surgical cavity F L	LEV, OXC
6	12	m	9y	Structural FLE	CPS	Caveroma F R	none
7	9	m	4y	Structural FLE	SPS/CPS	Extensive R polymicrogyria	LEV, VPA
8	28	f	11y	FLE of unclear origine	SPS/CPS GTCS	Unclear lesion F R, including insular cortex.	LTG, LCM
9	71	m	70y	Structural TLE	CPS	Cystic tumor mesio-temporal L	VPA
10	31	f	31y	TLE	CPS	Normal	OXC
11	60	m	40y	Structural TLE	CPS	Defect /sclerosis T pole L.	OXC
12	23	m	14y	Structural TLE	SPS, CPS	FCD T L	LTG
13	40	f	23y	Bilateral TLE	CPS, GTCS	Malrotation HC R	LTG
14	14	f	7y	Structural FLE	CPS	FCD F R	VPA, OXC
15	16	m	1y	Structural PLE	CPS	Tuberous sclerosis	LEV, ZNS

*AED, antiepileptic drugs; C, central; CPS, complex partial seizures; FCD, focal cortical dysplasia; f, female; F, frontal; FLE, frontal lobe epilepsy; GTCS, generalized tonic clonic seizures; HC, hippocampus; m, male; L, left; LCM, lacosamide; LEV, levetirazetam; LTG, lamotrigine; O, occipital; OXC, oxcarbazepin; P, parietal; PLE, parietal lobe epilepsy; R, right; SPS, simple partial seizures; y, years; T, temporal; TLE, temporal lobe epilepsy; VPA, valproate; ZNS, zonisamide*.

### Occurrence of negative BOLD responses

Details about all IED types and resulting BOLD responses are given in Table 2. The average size of AAL DMN template for each individual patient was 182.5 ± 14.9 cm^3^. The average size of brain areas showing significant negative BOLD responses was 109.2 ± 96.5 cm^3^. The average overlap between both areas was 12.3 ± 14.3 cm^3^. The large variation in overlap mainly results from the strongly varying amount of negative BOLD seen in different patients and distinct IED types.

**Table 2:** **Summary of IED types and overlap between DMN and negative BOLD**.

**Patient #**	**IED type**	**IED topography**	**Location T vs. Ex-T**	**# of IED**	**% of neg BOLD in DMN**	**% of DMN covered by neg. BOLD**
1	1	TP right	T	6	11	6
2	2	F T right	T	4	12	14
	3	T right	T	1	19	9
3	4	F right	EX-T	7	10	5
	5	PO Left	EX-T	2	15	6
	6	F left	EX-T	13	11	3
4	7	T right	T	1	18	4
	8	P right	Ex-T	2	21	7
	9	F right	Ex-T	3	9	6
5	10	C left	Ex-T	46	17	5
	11	T left	T	8	16	32
	12	F pole left	Ex-T	9	10	2
	13	F right	Ex-T	3	10	4
6	14	T left	T	2	12	7
7	15	F pole right	Ex-T	1	0	0
	16	FP right	Ex-T	1	24	2
8	17	F left	Ex-T	3	3	2
	18	FP right	Ex-T	21	4	5
	19	T right	T	2	5	0
9	20	F pole left	Ex-T	2	6	5
	21	F left	Ex-T	2	6	4
	22	T right	T	2	3	2
10	23	T right	T	1	6	2
11	24	FT left	T	2	10	5
	25	CP right	Ex-T	1	7	0
12	26	FT left	T	10	10	14
13	27	T right	T	3	15	29
	28	P right	Ex-T	12	14	2
14	29	F pole right	Ex-T	1	21	13
15	30	F pole right	Ex-T	4	8	5
Mean and SD					11.2 ± 6.2	6.7 ± 8

*C, central; DMN, default mode network; Ex-T, extra-temporal; F, frontal, IED, interitcal epileptic discharge; P, parietal; pos, positive; neg, negative; SD, standard deviation; T, temporal*.

**Figure 1:**
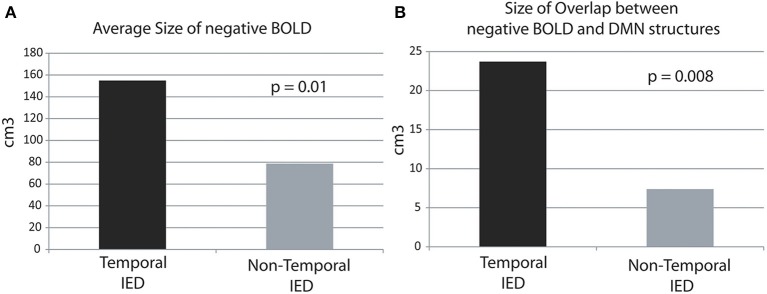
**Average size of overlap in cubic-centimeters between the negative BOLD and DMN structures in temporal and extra-temporal IED**. The average size of negative BOLD as well as the overlap is significantly larger for temporal than extra-temporal IED. **(A)** Average size areas covered by negative BOLD.

The average size of negative BOLD in temporal IED was with 154.9 ± 126.1 cm^3^ was significantly larger than in extra-temporal with 78.7 ± 56.4 cm^3^ (*p* = 0.01, Figure 1A). The average size of overlap between both regions was significantly larger in temporal IEDs with 23.7 ± 19.6 cm^3^ than for extra-temporal IEDs with 7.4 ± 5.1 cm^3^ (*p* = 0.008, Figure 1B).

### Percentage of overall negative BOLD responses found in DMN

**Figure 2:**
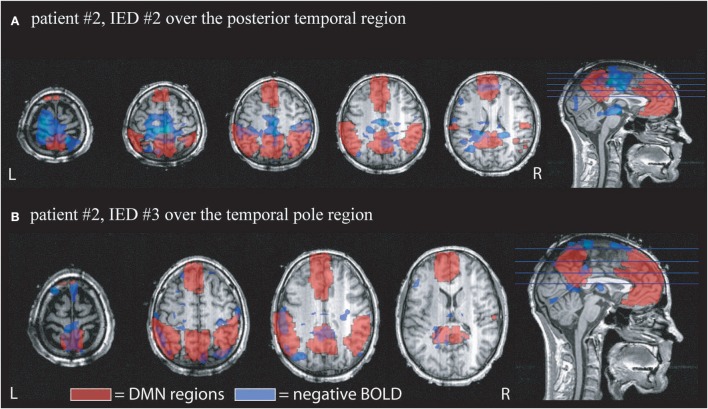
**Example of the overlap between negative BOLD responses and DMN regions**. Patient # 2 had two different IED generated over the anterior and posterior region of temporal structures. DMN regions according to the AAL atlas are shown in red, negative BOLD in blue. **(A)** Shows the overlap for IED # 2 which occurred 4 times during the scan. 12% of the negative BOLD was located within the DMN and 14% of the DMN were covered by negative BOLD. **(B)** Shows the response to IED # 2 which only occurred once during the scan time. 19% of negative BOLD was located within DMN regions and 9% of DMN was covered by negative BOLD.

**Figure 3:**
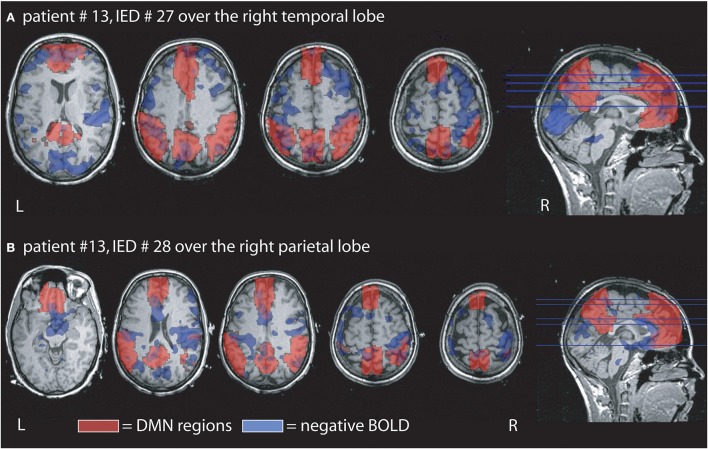
**Example of the overlap between negative BOLD responses and DMN regions**. Patient # 13 had two different IED one generated over the right temporal and the other over the right parietal region. **(A)** Demonstrates that overlap for the temporal IED with 15% of the negative BOLD located within the DMN and 29% of the DMN covered by negative BOLD. In agreement with our findings the overlap is larger than in the parietal IED of the same patient even if the temporal IEDs only occurred 3 times, while the parietal time was seen 12 times during the measurement. **(B)** Shows the negative BOLD changes related to the parietal IED. 14% of the negative BOLD are located in the DMN region and 2% of the DMN are covered by negative BOLD.

The average percentage of overall negative BOLD responses found within the DMN structures was 11.2 ± 6.1%. Again a large variation was seen between IED types. Seventeen patients showed between 10 and 20% overlap and 3 patients more than 20% overlap (see Table 2, for examples see Figures 2, 3).

**Figure 4:**
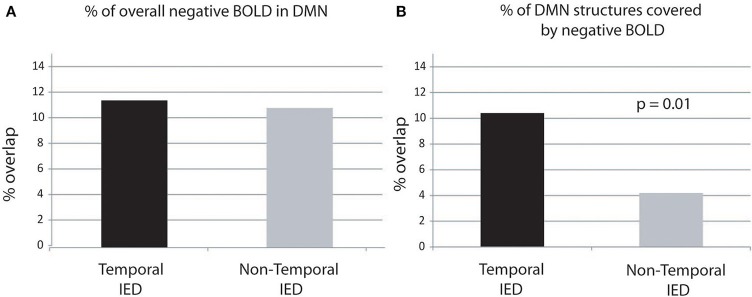
**Percentage of overlap between negative BOLD and DMN structures**. The percentage of DMN structures covered by negative BOLD is significantly higher in temporal than extra-temporal IED. Panel **(A)** display of the negative BOLD lying within regions of the DMN. Panel **(B)** display of the percentage of DMN covered by negative BOLD.

There was no significant correlation between the number of single IED per IED type and the amount of overlap. There was no significant difference between IEDs of temporal (11.5 ± 5%) and extra-temporal origin (10.9 ± 6.6%) (Figure 4A).

### Percentage of DMN covered by negative BOLD:

The average percentage of DMN structures covered by negative BOLD was 6.7 ± 7 cm^3^. Three patients showed between 10 and 20% overlap and two patients more than 20% overlap (see Table 2, for examples see Figures 1, 2).

There was no significant correlation between the number of IEDs per IED type and the amount DMN structures covered by negative BOLD. There was a significantly larger area of DMN structures covered by negative BOLD for IED with temporal origin (10.4 ± 10.3%) that IED of extra-temporal origin (4.2 ± 2.9%, *p* = 0.01) (Figure 4B).

### Comparison of temporal and non-temporal IEDs

Six patients had both temporal and extra-temporal IEDs (for details see Table 2), in one patient three comparisons, in three patients two comparisons and in two patients one comparison between temporal and extra-temporal IEDs were possible. In regard to the percentage of DMN structures covered by negative BOLD the statistical comparison just within the single patients shows a trend toward temporal IED causing larger overlap than extra-temporal ones (*p* = 0.07). For the percentage of negative BOLD lying within DMN structures no significant difference can been seen as for the overall group (*p* = 0.2).

## Discussion

The present study confirms the observation that a high number of patients with focal epilepsy show alterations in the DMN during focal IED occurrence. This observation is mainly possible due to the use of MREG which increases the sensitivity of event-related fMRI for IEDs. The amount of negative BOLD in the DMN was highly variable between distinct types of IED and stronger in IEDs generated in the temporal lobe. This suggests that different IEDs may affect attention and consciousness to variable degrees and it may be of clinical interest for patients with epilepsies to identify those subtypes with a large effect on important networks such as the DMN.

## Methodological issues

The present analysis of single patients was to a large extent only possible as a result of the increased sensitivity of the MREG sequence (Zahneisen et al., 2012; Assländer et al., 2013). During the analysis, statistical methods were carefully adapted to correct for the increase of autocorrelations and multiple comparisons. In EEG-fMRI in epilepsy, the definition of a gold standard to which all BOLD changes can be compared is difficult and the most valid is probably comparing BOLD changes with activity from intracranial EEG (Pittau et al., 2011) or surgical removal and postsurgical outcome (Thornton et al., 2010). However, BOLD changes detected by MREG have so far only been compared with the lobe of spike origin or the localization of lesion (Jacobs et al., 2014), as the method is still quite recent; additional data acquisitions will need to be performed to allow a valid comparison with other measures.

As with all EEG-fMRI studies, it is important to exclude sources of artifact which may result in incorrect BOLD responses. One advantage of MREG is the ability to measure un-aliased physiological artifacts such as respiration and ECG, which could then be corrected as part of our analysis (LeVan et al., 2012).

A second potential source for mistakes during EEG-fMRI is the false detection of motion artifacts such as the ballistocardiogram as IED (Flanagan et al., 2009; Jansen et al., 2012). The first step to avoid this mistake is the thorough correction of EEG artifacts, which has been performed with all currently available methods in the present study (Allen et al., 1998; Debener et al., 2007). Moreover, Van Houdt and coworkers could show that a more robust identification of IED results from reviewing the EEGs by more than one reviewer, as we performed in our analysis (Van Houdt et al., 2010) MREG increases sensitivity to a point where BOLD changes related to artifacts may also be more likely to be detected, which is why we felt that IED selection should be rather specific than sensitive.

In the present study IEDs occurring during the time window of the ballistocardiogram were not included in the analysis. This measure likely resulted in the exclusion of true IEDs and thus in a decreased sensitivity of the identified BOLD responses (Flanagan et al., 2009). However, this was considered a more benign effect compared to the potential inclusion of non-epileptiform motion events among true IEDs, which may result in not only a decreased sensitivity for the identification of IED-related BOLD responses, but also possibly causing spurious negative BOLD in the DMN (Flanagan et al., 2009). Due to the use of MREG as fMRI sequence and its high sensitivity for the detection of IED related BOLD changes (Jacobs et al., 2014), which may compensate the loss of sensitivity, as well as for the sake of our primary goal to analyze negative BOLD in DMN structures, it thus appears to be more reasonable to exclude events coinciding with movements such as the BCG. The development of better monitoring of patient motion is likely to greatly facilitate the distinction of true and motion-related epileptiform events (Masterton et al., 2007; Flanagan et al., 2009; LeVan et al., 2013).

### Origin of negative BOLD responses

It should be pointed out that we did not preselect patients in regard to whether they showed any negative BOLD for this analysis. Therefore, it is not surprising that the area covered by negative BOLD varied between zero and 380 cubic-centimeters depending on patient and spike type. The underlying physiological mechanism of negative BOLD is largely unknown. One theory has suggested that it might result from a “vascular steal” phenomenon, which implies that neighboring areas of increased blood flow and BOLD cause a decreased blood flow and negative BOLD (Harel et al., 2002). In previous as well as the current study this explanation seems rather unlikely as we could not observe any correlation between positive and negative BOLD as well as no spatial relationship (Kobayashi et al., 2006a; Jacobs et al., 2014). Other authors suggest that the neurovascular coupling necessary to observe the well-known positive BOLD effect might be impaired in some regions of patients with epilepsy, which could result in a lack of blood flow increase caused by high deoxyhemoglobin levels (Fink et al., 1996; Bruehl et al., 1998). A study of Stefanovic and colleagues however found convincing evidence for intact neurovascular coupling in patients with epilepsy (Stefanovic et al., 2005). Impaired neuro-vascular coupling is thus unlikely to explain the presence of negative BOLD changes either in the DMN or elsewhere, as described before as well as in our study.

Another possible explanation for the occurrence of negative BOLD might be a decreased neuronal activity at the time of IEDs in these regions. This explanation is in line with the observation of increased concentrations of the inhibitory transmitter GABA in regions of negative BOLD (Chatton et al., 2003; Stefanovic et al., 2004). If negative BOLD reflects increased inhibition, negative BOLD related to IEDs is suggestive of areas with increased inhibition associated with IED. While IEDs are considered excitatory phenomena, inhibition directly after the IED has often been described (Urrestarazu et al., 2006). This observation of postspike inhibition is in line with results from EEG-fMRI, in which negative focal BOLD changes in the epileptic focus were often preceded by a positive BOLD in the same area (Jacobs et al., 2009). Thus, negative BOLD could also been seen as an undershoot phenomenon or a post-spike period of inhibition. Whether this interpretation can explain negative BOLD in the DMN however is rather questionable as no earlier positive responses were observed in the same regions (Gotman et al., 2005; Jacobs et al., 2014). Nevertheless, one could imagine that DMN structures or the connections between them are inhibited during an IED. Independent of mechanisms of negative BOLD the most important finding of this and previous studies is that DMN structures can be related to IED occurrence and that this effect is largely variable.

### Interactions between default mode network and epilepsy

The DMN was originally discovered as a network of structures which are active or show positive BOLD during periods of rest in contrast to the actual activity under examination (Shulman et al., 1997; Mazoyer et al., 2001). Its functional role is not completely understood yet, but it is strongly activated during biographical memory retrieval, envisioning the future and conceiving the perspectives of others, while its activity is reduced during periods in which the brain focuses on external stimuli (Dosenbach et al., 2006). Changes in the structures and functional connectivity of the DMN as well as reduced activation of the network has been described in several brain diseases such as Alzheimer's dementia, schizophrenia and autism disorders (Greene et al., 2001; Lustig et al., 2003; Buckner et al., 2005; Fox et al., 2005). During inter-ictal and ictal epileptic activity, studies so far mostly reported negative BOLD changes in the DMN (Gotman et al., 2006; Laufs et al., 2007). Negative BOLD in the DMN in studies with healthy subjects have been interpreted as a decreased activity in the DMN when comparing tasks in which the DMN was highly active to those in which it was less active (Buckner et al., 2008). In line with this it has been hypothesized that the IEDs associated with negative BOLD in the DMN act like an external stimulus interrupting the function of the normal DMN (Cataldi et al., 2013). Fahoum and colleagues analyzed IED-related changes in the intracranial EEG in the DMN regions and observed a reduction of gamma power and increase of lower frequency EEG activity specific to these regions (Fahoum et al., 2013). Suppression in the gamma band is in line with brain activity usually observed after external stimulation such as visual stimuli (Ossandón et al., 2011). Thus, it seems that the IED have similar effects on the epileptic brain's DMN as repetitive external stimuli.

Our study confirmed previous observations that the degree to which IEDs affect the DMN is largely variable. While a clear correlation between cognitive decline and disruption of the DMN has been observed in Alzheimer's dementia (Broyd et al., 2009; Miao et al., 2011), the clinical importance of alterations in the DMN is still unknown in epilepsy. Some authors suggest that negative BOLD on the DMN directly reflects a short interruption of attention or consciousness during the epileptic events, while others suggest rather a long-term effect on cognition due to the repetitive interruption of the DMN by IEDs (Blumenfeld et al., 2004; Laufs et al., 2007; Fahoum et al., 2012).

The first hypothesis would suggest a short repetitive interruption of consciousness or normal function of the region generating the IED. Such loss of consciousness is especially observed in generalized epilepsies where trains of generalized spikes and waves lead to interrupted consciousness during absence seizures. These seizures are one of the very few seizure types which can be observed during EEG-fMRI as they occur frequently and without excessive motion. Absence seizures are associated with strong negative BOLD changes in the DMN (Blumenfeld, 2005; Moeller et al., 2008). Ictal EEG-fMRI in focal epilepsy is rare and only a few reports demonstrated DMN changes during these seizures (Blumenfeld and Taylor, 2003). Again during these focal seizures, reduction in DMN activity was mainly observed when loss of consciousness or secondary generalization occurred (Norden and Blumenfeld, 2002; Blumenfeld et al., 2009). EEG studies support this observation by showing neocortical slowing during the period of impaired consciousness in TLE (Blumenfeld et al., 2004; Englot et al., 2010). The association between decreased consciousness and DMN changes observed during ictal activity might also be true for focal IEDs with the only difference that focal IEDs are usually generated over too small brain areas to cause clinically visible alterations of consciousness.

Short impairments of cognitive function as described in TCI (Binnie, 2003) can only be detected using complex cognitive testing and do not refer to consciousness levels in general but the specific task at hand. Existing studies are biased like ours toward patients with frequent IEDs and limited to short test periods. However, it could be clearly shown that temporal, more precisely hippocampal, IEDs can lead to transient cognitive impairment by disrupting memory maintenance and retrieval (Kleen et al., 2013). While cognitive interruption during working memory has been associated with negative BOLD in the DMN during generalized IEDs (Chaudhary et al., 2013), it remains unclear whether transitory cognitive impairment related to focal, specifically temporal, IEDs would result in negative BOLD changes. Thus, the strongest evidence for the idea that negative BOLD in DMN reflects short interruptions of cognition or even consciousness comes from the observation that the DMN changes were stronger in TLE, which is usually associated with earlier and stronger impairment of consciousness than neocortical epilepsies (Laufs et al., 2007; Cataldi et al., 2013).

The rare and scarce occurrence of DMN changes in the analysis of single patients due to the low sensitivity of classical fMRI sequences has been a major challenge for such a study design. The fact that we saw negative BOLD changes in the DMN in all patients suggests that MREG will facilitate this type of investigation and hopefully enable us to answer the question whether focal IED have the potential to interrupt consciousness or cognition.

The alternative hypothesis suggests that negative BOLD in the DMN reflects negative long-term effect of IED on cognition. This would mean that IEDs associated with DMN changes reflect stronger long-term interference with cognition than IEDs which do not deactivate the DMN. Again this question could only be answered by having long-term studies correlating DMN deactivation and cognitive decline in patients with epilepsy, as has been done for Alzheimer's dementia (Broyd et al., 2009; Miao et al., 2011). In our study most patients had long-lasting refractory epilepsy as they were recruited from an epilepsy center specialized in pre-surgical diagnostics and epilepsy surgery. Cognitive decline and loss of specific cognitive function are well-known disabilities in epilepsy and recurrent uncontrolled seizure are correlated with more severe impairments (Carreño et al., 2008; Avanzini et al., 2013). It might therefore be that the frequent occurrence of negative BOLD of DMN network reflects the fact that most of our patients had more severe epilepsy and cognitive disability than the average population of patients with epilepsy. Nevertheless, most studies using EEG-fMRI in epilepsy are performed in large epilepsy centers and pre-surgical units (Moeller et al., 2009; Thornton et al., 2010). Thus, the frequent occurrence of negative BOLD in DMN is probably mostly reflecting increased sensitivity of MREG and again studies correlating cognitive performance and strength of DMN disruption are necessary to understand the clinical importance of our findings. As EEG-MREG is a non-invasive tool it might be even more interesting to investigate patients with new onset focal epilepsy to see whether changes in DMN during IED are prognostic for cognitive problems in these patients.

### Effect of frequent spiking

IED are often believed to reflect the epileptic potential of the underlying tissue and are usually monitored in EEG recordings to assess treatment control. While it is certainly true that frequent inter-ictal activity is associated with severity of disease and cognitive decline in some epileptic syndromes such as continuous spike wave status (CSWS) in sleep (Pera et al., 2013), for most epilepsies there is no clinical correlation between frequency of IED and seizure frequency or cognitive decline (Spencer et al., 2008). Nevertheless, one could imagine that a frequent occurrence of IED disturbs the brain networks more extensively than rare occurrence, resulting in more prominent negative effects on attention and cognition. Our study did however not suggest any correlation between IED frequency and DMN changes. It has to be noted that our study was biased toward patients with regular IED occurrence on the EEG, as this was a necessary requirement for successful EEG-fMRI analysis in the time-limited framework of MR scanning. It is therefore not possible to draw conclusion about patients in whom rare or no IEDs are seen on the scalp EEG. Still IED numbers varied quite strongly between 1 and 46 IED within the 40-min measurement and no evidence was found suggesting that patients with high IED numbers showed more prominent DMN involvement. This suggests that DMN involvement is not dependent on the acute likelihood of epileptic tissue to generate spikes but rather on the anatomical structure generating the IED and its connections with other brain regions.

### DMN involvement during temporal and neocortical IEDs

It was the aim of the present study to investigate the overlap between DMN and negative BOLD occurrence. As we hypothesized this overlap was larger in temporal than neocortical IEDs, as has been shown in previous group analysis (Laufs et al., 2007). Changes in the DMN in TLE have been investigated quite extensively. It seems unclear whether these are more of a structural or functional nature. The hippocampal formation itself is not only the generator of IEDs but also sometimes considered to be part of the DMN and it often shows extensive structural damage as part of the epilepsy syndrome. Thus, it has been debated whether reduced connectivity and activity of DMN might be a result of these structural changes rather than reflecting an ongoing interruption of DMN function (Liao et al., 2010, 2011). However, functional studies like ours which find negative BOLD at the time of IEDs in contrast to baseline activity underline the suggestion that the DMN undergoes a continuous functional alteration. The same is suggested by studies of functional connectivity which not only discovered decreased connectivity between DMN nodes but also hyper-connectivity between others in TLE (Zhang et al., 2010; Pittau et al., 2012).

Recently TLE is increasingly believed to be a network disease and permanent changes such as atrophy of brain regions have been described even far from the focus (Spencer, 2002; Bartolomei et al., 2005, 2008). EEG-fMRI analysis of patients with TLE support this observation, as positive as well as negative BOLD responses are rarely focal but often suggest involvement of subcortical and distant cortical regions (Laufs et al., 2007; Kobayashi et al., 2009). The present study focused on the analysis of negative BOLD responses and it is therefore remarkable that not only the overlap between DMN structures and negative BOLD was larger in temporal spikes but it was also the case for the overall extent of negative BOLD. Indeed, while the initial focus of the study was to investigate deactivations in DMN structures associated with IEDs, the large majority of negative BOLD responses actually occurred outside DMN regions. This finding might be interpreted as an indication that IED generated over temporal regions affect larger networks by fast propagation and that this effect might be predominantly inhibitory. Moreover not only the DMN but also other resting state networks such as the attention network and executive control network have been described to be impaired in TLE (Cataldi et al., 2013). The lack of significant difference in the percentage of negative BOLD in DMN regions between temporal and extratemporal spikes also indicates that deactivations are not necessarily specific to the DMN, and additionally may suggest a large variability within the temporal and extra-temporal groups. Therefore, our study suggests that additional investigation of the localization of negative BOLD responses outside the DMN might reveal other resting state that show interference by focal IEDs not only in TLE patients in general, but also at the individual patient level.

## Conclusion

In the present study quantification of overlap between DMN regions and negative BOLD occurrence after focal IED revealed involvement of DMN structures to varying extent in all patients. MREG as a method of fast fMRI allows very sensitive detection of BOLD changes in the DMN structures. Interestingly the frequency of IEDs did not affect the occurrence of negative BOLD in the DMN, but the origin of IED did. Thus, preexisting network structures seem to be a relevant factor for the ability of an IED to interfere with the DMN. IED generated from the mesial temporal structures which are part of the DMN or their vicinity result in stronger interruption of the DMN activity. The clinical implications of these findings are unknown, but if spontaneous repetitive IEDs interrupt the resting state networks of the brain in similar but less directed way as external stimuli, a resulting impairment of consciousness and cognition are likely.

### Conflict of interest statement

The authors declare that the research was conducted in the absence of any commercial or financial relationships that could be construed as a potential conflict of interest.

## Abbreviations

BOLD: Blood Oxygenation Level Dependent
DMN: default mode network
EPI: Echo Planar Imaging
fMRI: functional magnetic resonance imaging
IED: inter-ictal epileptic discharge
MREG: Magnetic-Resonance-Encephalography
TLE: Temporal Lobe Epilepsy.
